# The selenoenzyme type I iodothyronine deiodinase: a new tumor suppressor in ovarian cancer

**DOI:** 10.1002/1878-0261.13612

**Published:** 2024-03-01

**Authors:** Adi Alfandari, Dotan Moskovich, Avivit Weisz, Aviva Katzav, Debora Kidron, Mario Beiner, Dana Josephy, Aula Asali, Yael Hants, Yael Yagur, Omer Weitzner, Martin Ellis, Gilad Itchaki, Osnat Ashur‐Fabian

**Affiliations:** ^1^ Translational Oncology Laboratory Hematology Institute, Meir Medical Center Kfar Saba Israel; ^2^ Department of Human Molecular Genetics and Biochemistry, Faculty of Medicine Tel Aviv University Israel; ^3^ School of Medicine, Faculty of Medical and Health Sciences Tel Aviv University Israel; ^4^ Department of Pathology Meir Medical Center Kfar Saba Israel; ^5^ Division of Gynecologic Oncology Meir Medical Center Kfar Saba Israel; ^6^ Department of Obstetrics and Gynecology Meir Medical Center Kfar Saba Israel

**Keywords:** deiodinase, expression, gene silencing, ovarian cancer, proliferation

## Abstract

The selenoenzyme type I iodothyronine deiodinase (DIO1) catalyzes removal of iodine atoms from thyroid hormones. Although DIO1 action is reported to be disturbed in several malignancies, no work has been conducted in high‐grade serous ovarian carcinoma (HGSOC), the most lethal gynecologic cancer. We studied *DIO1* expression in HGSOC patients [The Cancer Genome Atlas (TCGA) data and tumor tissues], human cell lines (ES‐2 and Kuramochi), normal Chinese hamster ovarian cells (CHO‐K1), and normal human fallopian tube cells (FT282 and FT109). To study its functional role, *DIO1* was overexpressed, inhibited [by propylthiouracil (PTU)], or knocked down (KD), and cell count, proliferation, apoptosis, cell viability, and proteomics analysis were performed. Lower DIO1 levels were observed in HGSOC compared to normal cells and tissues. TCGA analyses confirmed that low *DIO1* mRNA expression correlated with worse survival and therapy resistance in patients. Silencing or inhibiting the enzyme led to enhanced ovarian cancer proliferation, while an opposite effect was shown following *DIO1* ectopic expression. Proteomics analysis in *DIO1*‐KD cells revealed global changes in proteins that facilitate tumor metabolism and progression. In conclusion, DIO1 expression and ovarian cancer progression are inversely correlated, highlighting a tumor suppressive role for this enzyme and its potential use as a biomarker in this disease.

## Graphical abstract

The selenoenzyme deiodinase type 1 (DIO1) has anti‐tumor actions in high‐grade serous ovarian cancer. Reduced levels of DIO1 in HGSOC cells and human tissues facilitate proliferation and may promote tumorigenesis in this aggressive disease.

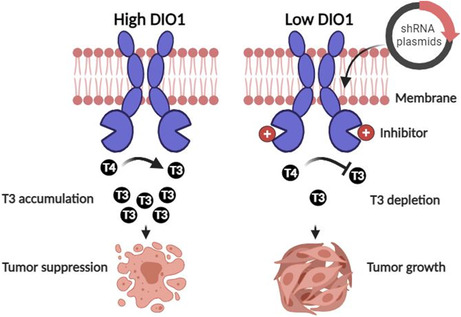

**Abbreviations**
AKAP: A‐kinase anchoring protein family members
CHO‐K1: Chinese hamster ovarian cells
COA6: cytochrome c oxidase assembly factor 6
DIO1: type I iodothyronine deiodinase
DIO2: type II iodothyronine deiodinase
DIO3: type III iodothyronine deiodinase
ECL: enhanced chemiluminescence
FC: flow cytometry
FFPE: formalin‐fixed paraffin‐embedded
FT: fallopian tube
GAPDH: glyceraldehyde 3‐phosphate dehydrogenase
H&E: hematoxylin and eosin
HGSOC: high‐grade serous ovarian carcinoma
HK1: hexokinase 1
HRP: horseradish peroxidase
IHC: immunohistochemistry
KD: knocked down
MFI: mean fluorescence intensity
panther
: Protein Analysis Through Evolutionary Relationships
PDHA1: pyruvate dehydrogenase E1 subunit alpha 1
pERK: phosphorylated ERK
PKM2: pyruvate kinase 2 (PKM2)
PRIM1: DNA primase polypeptide 1
PTU: propylthiouracil
SC: subcutaneously
SEC: selenocysteine
STIC: serous tubal *in‐situ* carcinoma
TCGA: The Cancer Genome Atlas
TH: thyroid hormone
TR: thyroid receptors
TRXR1: thioredoxin reductase 1
WB: Western blots

## 1 Introduction

Epithelial ovarian cancer is a gynecological malignancy that, although around one‐tenth as common as breast cancer, is associated with a disproportionately high mortality rate. While the cure rate in women with the disease localized exclusively to the ovary at the time of diagnosis is high [1, 2], the majority of patients are diagnosed after the disease has metastasized. At these advanced stages, despite improvement in diagnostic and therapeutic tools, the 5‐year survival rate is still less than 50%. High‐grade serous ovarian carcinoma (HGSOC) is the most common advanced stage tumor. Current dogma posits that HGSOC originates, in most cases, from serous tubal *in‐situ* carcinoma (STIC) in the patients' fallopian tube epithelium, in which p53 gene mutations occur early in carcinogenesis [3, 4].

Fast‐growing tumor tissues are dependent on deiodinase enzymes, which belong to the selenoprotein family [5]. The catalytic core in these enzymes contains the essential trace element selenium (Se), in the form of the 21st amino acid, selenocysteine (Sec) [6]. All deiodinases are thyroid hormone (TH)‐regulating enzymes via deiodination of inner or outer ring iodine atoms in the hormone structure [7, 8, 9, 10]. The family consists of three members, type I iodothyronine deiodinase (DIO1) and type II (DIO2), which initiate TH action by converting T4 into the biologically active hormone triiodothyronine (T3), and type III iodothyronine deiodinase (DIO3), which converts T4 and T3 into inactive metabolites. DIO1 can also, under some conditions, deactivate (5‐deiodination) TH metabolites. DIO1 and DIO3 are membrane‐anchored proteins, whereas DIO2 resides at the endoplasmic reticulum. Together, the DIO family fine‐tunes the intracellular bioavailability of T3 on the basis of tissue‐specific and functional demands.

T3 regulates a wide range of cellular processes via its nuclear thyroid receptors (TRs) and plays a vital role in development, tissue differentiation, and maintenance of cell metabolic balance [11]. This delicate and dynamic equilibrium between cell proliferation and differentiation suggests a possible role for the deiodinase enzymes in tumorigenesis via regulation of intracellular T3 levels. Previous studies have suggested that the expression of DIO1 is dysregulated in various tumors [11, 12, 13]; however, none have comprehensively analyzed this unique protein in HGSOC. In this study, we have identified a unique expression pattern of DIO1 in ovarian cancer cells and tissues and provided evidence, by several complementary methods, suggesting that this enzyme has tumor‐suppressive activities.

## 2 Materials and methods

### 2.1 Reagents and chemicals

Propylthiouracil (PTU) was purchased from Sigma‐Aldrich (St. Louis, MO, USA). Antibody list is presented in Table S1.

### 2.2 Cell lines

Human HGSOC cells ES‐2 (CVCL_3509) were purchased from the ATCC. Kuramochi (CVCL_1345) and normal immortalized fallopian tube cells (FT282/CVCL_A4AX and FT109) were provided by Dr. Ruth Perets (Rambam Medical Center, Haifa, Israel). Normal Chinese hamster ovarian cells (CHO‐K1) were a kind gift from Prof. Philippe Clézardin (University of Lyon, Lyon, France). CHO‐K1 and the HGSOC cells were grown in complete RPMI1640 medium, supplemented with 10% heat‐inactivated FBS and 1% penicillin–streptomycin antibiotics. FTs were grown in DMEM F‐12 medium (Biological Industries, Beit HaEmek, Israel). STR/mutation profiling was conducted in the past 3 years for cell authentication, and mycoplasma was screened periodically.

### 2.3 DIO1 knockdown

Stable transfections with shRNA for DIO1 (sc‐77146‐SH, Santa Cruz Technologies, Dallas, TX, USA) were performed following the manufacturer's instructions using shRNA plasmid transfection reagent (sc‐108061, Santa Cruz Technologies). GFP plasmid was used as a positive control (sc‐108083, Santa Cruz Technologies), and nonspecific mock shRNA plasmid (scrambled) was used as negative control (sc‐108060, Santa Cruz Technologies). After transfections and puromycin selection, stable DIO1‐KD ES‐2 clones were isolated. The successful inhibition of DIO1 was confirmed at the RNA level (real‐time PCR) and protein levels (Western blot).

### 2.4 Transient DIO1 overexpression

Transient transfections with DIO1 expression plasmid (DIO1‐PCDNA3, a generous gift from Prof. Piekielko‐Witkowska, Department of Biochemistry and Molecular Biology Centre of Postgraduate Medical Education, Warsaw, Poland) were conducted using Mirus TransIT‐X2 transfection reagent (Mirus Bio Technologies, Madison, WI, USA). An empty pcDNA3 vector was used as a negative control. The transient DIO1 overexpression was validated by Western blot.

### 2.5 RNA extraction, cDNA, and reverse transcription

RNA was extracted using NucleoSpin RNA II kit (Macherey‐Nagel, Düren, Germany) and eluted in 40 μL RNase‐free water. RNA concentration and purity were measured using NanoDrop™ 1000 Spectrophotometer (Thermo Scientific, Wilmington, DE, USA). RNA (200 ng) was reverse‐transcribed using High Capacity cDNA Reverse Transcription Kit (Applied Biosystems, Carlsbad, CA, USA), according to instructions.

### 2.6 Real‐Time PCR

RNA was reversed‐transcribed, and cDNA was analyzed using SYBR Green/7500 Fast system (Applied Biosystems). Primers for DIO1 were designed (Primer‐Express software) in different exons in order to minimize DNA contamination. DIO1 forward primer‐CACTGCCTGAGAGGCTCTACAT. DIO1 reverse primer‐CCAGAACAGCACGAACTTCCTC. β‐actin forward primer: CCTGGCACCCAGCACAAT. Reverse primer: GCCGATCCACACGGAGTACT. The comparative threshold cycle method (2‐ΔΔCT), after normalization to actin, was used for relative quantifications.

### 2.7 Flow cytometry (MACSQuant, Miltenyi Biotec, Bergisch Gladbach, Germany)

DIO1 levels: Cells were harvested and labeled with the fluorescently conjugated antibodies (1 μg/2 × 10^5^ cells) detailed in Table S1. Absolute cell counts: Cells were collected in PBS and counted. Cell cycle: Cells were harvested, fixed using cold 70% ethanol, centrifuged, stained with DNA propidium iodide (PI; 50 mg·mL^−1^)/RNAse A (10 mg·mL^−1^; Sigma‐Aldrich), and analyzed. Cell death: Cells were incubated with 10 μL Annexin V (FITC conjugated)/5 μL PI and analyzed by FACS (Annexin+/PI−, early apoptosis; Annexin+/PI+, late apoptosis/necrosis).

### 2.8 PrestoBlue cell viability reagent

PrestoBlue (A13261; Invitrogen, Carlsbad, California, USA) was added to cell supernatant (10 μL per well) and incubated at 37 °C for 30 min and read using microELISA reader at 595 nm.

### 2.9 CyQuant cell proliferation assay

CyQuant (C35011; Invitrogen) was added to cells (100 μL per well) and incubated at 37 °C for 60 min and read using microELISA reader at 535 nm.

### 2.10 Western blotting

Whole cell lysates were separated using lysis buffer containing beta‐glycerophosphate, sucrose, EDTA, EGTA, sodium metavanadate, sodium diphosphate decahydrate, and Triton, on 12.5% polyacrylamide gels, transferred to PVDF, and analyzed by Western blots using the DIO1 antibodies mentioned above. Visualization was performed as previously described [14] using horseradish peroxidase (HRP)‐conjugated secondary antibody (1:10 000) followed by enhanced chemiluminescence (ECL) detection (ECL kit; Millipore, Merck, Darmstadt, Germany). Integrated optical densities of bands were measured (Image reader Las3000), multi‐gauge v3.0 software, and normalized to protein loading.

### 2.11 Proteomics

For total proteome, proteins were subjected to proteolysis, digested by trypsin as previously described [14]. The tryptic peptides were desalted using C18 tips, dried, and resuspended in 0.1% formic acid. The peptides were analyzed by reversed‐phase capillary chromatography coupled online to tandem mass spectrometry (uLC‐MS/MS). Identified peptides were filtered with high confidence, top rank, mass accuracy, and a minimum of two peptides. High confidence peptides have passed the 1% false discovery rate (FDR) threshold (estimated false positives in a list of peptides). Semi‐quantification was done by calculating the peak area of each peptide. Keratins were filtered out since they might be a contamination that origins from dust, hair, and skin. Protein enrichment analysis and classification according to molecular functions, cellular localizations, and classes were performed using the Protein Analysis Through Evolutionary Relationships (panther) tool [15]. Protein–protein interaction network was generated using string v. 10.5 with default settings (minimum required interaction score: medium confidence 0.4) [16]. Bionic visualization (BionicVis) tool was used to generate proteomaps showing color‐coded quantitative composition of proteomes [17].

### 2.12 Immunohistochemistry (IHC)

Four micron sections were cut from archived formalin‐fixed paraffin‐embedded (FFPE) tissue blocks at the Meir Medical Center Pathology Department. Tissues were collected between January 2018 and February 2019 upon written informed consent, in compliance with the Meir Medical Center Institutional Review Board approval (#0305‐16‐MMC), in accordance with the Declaration of Helsinki. HGSOC diagnosis (grade 3–4) was confirmed by a certified pathologist based on clinical and pathological characteristics. IHC stains for DIO1, DIO3, p53, p16, pax8, and Ki67 were carried out as previously described [14] using the antibodies detailed in Table S1, on a Ventana Benchmark XT automatic stainer (Ventana, Tucson, Arizona, USA). Images were obtained by a microscope equipped with a camera with Olympus model BX41 for IHC slides and model IX71 for cell cultures (Olympus, Tokyo, Japan). Analysis was performed by cellSens Entry Olympus imaging software.

### 2.13 TCGA data

Analysis of the expression of DIO1 was extracted from The Cancer Genome Atlas (TCGA) database using the Kaplan–Meier Plotter [18] and the ROC Plotter tools [19].

### 2.14 HGSOC xenograft model

Nine‐week‐old female athymic nude mice (*n* = 3, Envigo, Ness‐Ziona, Israel) were maintained under specific pathogen‐free conditions and housed under controlled conditions (temperature: 20–24 °C; humidity: 60–70%). The mice housed in a cage under conventional conditions and fed chow and water *ad libitum* and allowed to acclimate for 1 week prior to their use according to study protocols. Mice were subcutaneously injected with scrambled control ES‐2 cells (1 × 10^6^ cells each) into the right flank. Twenty‐eight days later, tumors were collected for FFPE preparations and evaluated for DIO1 protein staining. The study was outsourced (Almog Diagnostic LTD, Shoham, Israel) following approval of Institutional Animal Care Committee (IACUC# 59‐08‐2018), in compliance with the recommendations of the Guide for Care and Use of Laboratory Animals.

### 2.15 Statistical analysis

Experiments were conducted at least three separate times in triplicates and analyzed for significance (*P* < 0.05) with Student's unpaired *t* test, Mann–Whitney test, or by ANOVA for multiple comparisons.

## 3 Results

### 3.1 DIO1 expression is lower in HGSOC cells compared to normal cells

**Fig. 1:**
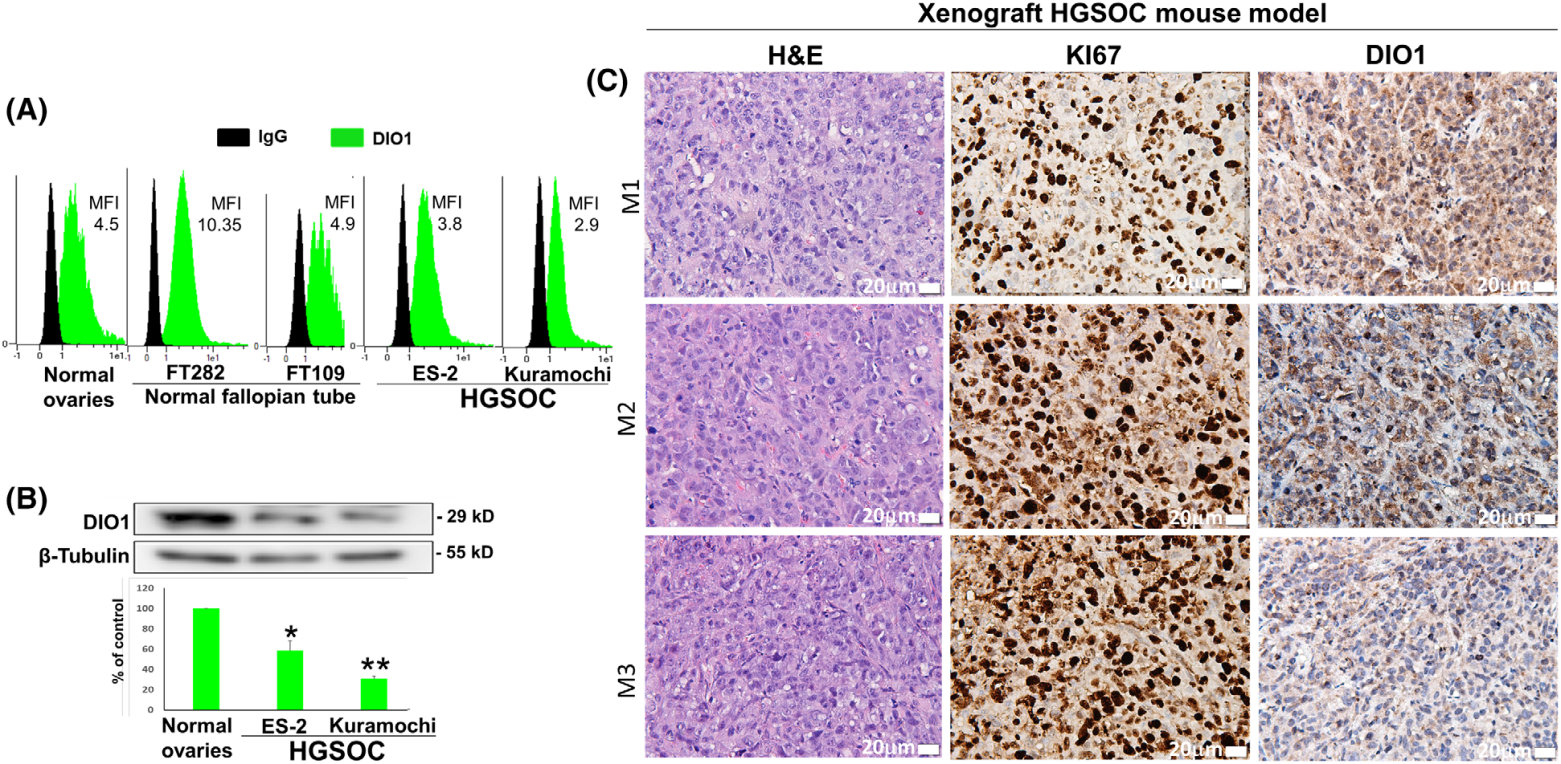
Low selenoenzyme type I deiodinase (DIO1) levels in high‐grade serous ovarian cancer (HGSOC) cell lines and xenografts. CHO‐K1 (normal ovaries), FT282, FT109, ES‐2, and Kuramochi cells were analyzed for the DIO1 by (A) flow cytometry and (B) Western blots. A representative analysis of three experimental repeats is presented. β‐tubulin was used for protein loading. Average DIO1 protein expression ± SEM is presented. *P < 0.05 and **P < 0.05 by ANOVA for multiple comparisons. (C) Hematoxylin and eosin (H&E) and immunohistochemistry (IHC) staining for DIO1 and KI67 in formalin‐fixed paraffin‐embedded (FFPE) tumors from three mice. Scale bar, 20 μm. Matched isotype control is presented in Fig. S2A. 20× objective, Olympus microscopy (Olympus, Tokyo, Japan).

The basal levels of DIO1 were analyzed in HGSOC cells (ES‐2 and Kuramochi), which were shown to correlate with the genomic profiling of ovarian cancer patients [20, 21]. The expression was compared with that of normal immortalized ovarian cells (CHO‐K1) and fallopian tube cells (FT282 and FT109). Flow cytometry (FC) analysis, using AF‐647‐tagged DIO1 antibody, indicated that the ovarian cancer cells express lower DIO1 protein levels compared to the normal cell models (Fig. 1A). The calculated mean fluorescence intensity (MFI) relative to isotype control (IgG) was 4.3 for CHO‐K1, 10.35 for FT282, 4.9 for FT109, and 3.39 and 2.78 for ES‐2 and Kuramochi, respectively. Similarly, DIO1 protein levels by Western blots (WB) were lower in the HGSOC cells (Fig. 1B). DIO1 antibody specificity was confirmed by several approaches (Fig. S1). Next, we wanted to assess whether DIO1 expression in the HGSOC cells will remain low when inoculated into mice or may be affected by the microenvironment *in vivo*. To that end, 1 × 10^6^ ES‐2 cells were inoculated subcutaneously (SC) into the flank of nude female mice (*n* = 3). In accord with the FC and WB data, we observed at study end (day 28) limited DIO1 IHC staining in the excised tumors (Fig. 1C). Isotype control is presented in Fig. S2A.

### 3.2 DIO1 positively correlates with better survival and is differentially expressed in HGSOC tumors

**Fig. 2:**
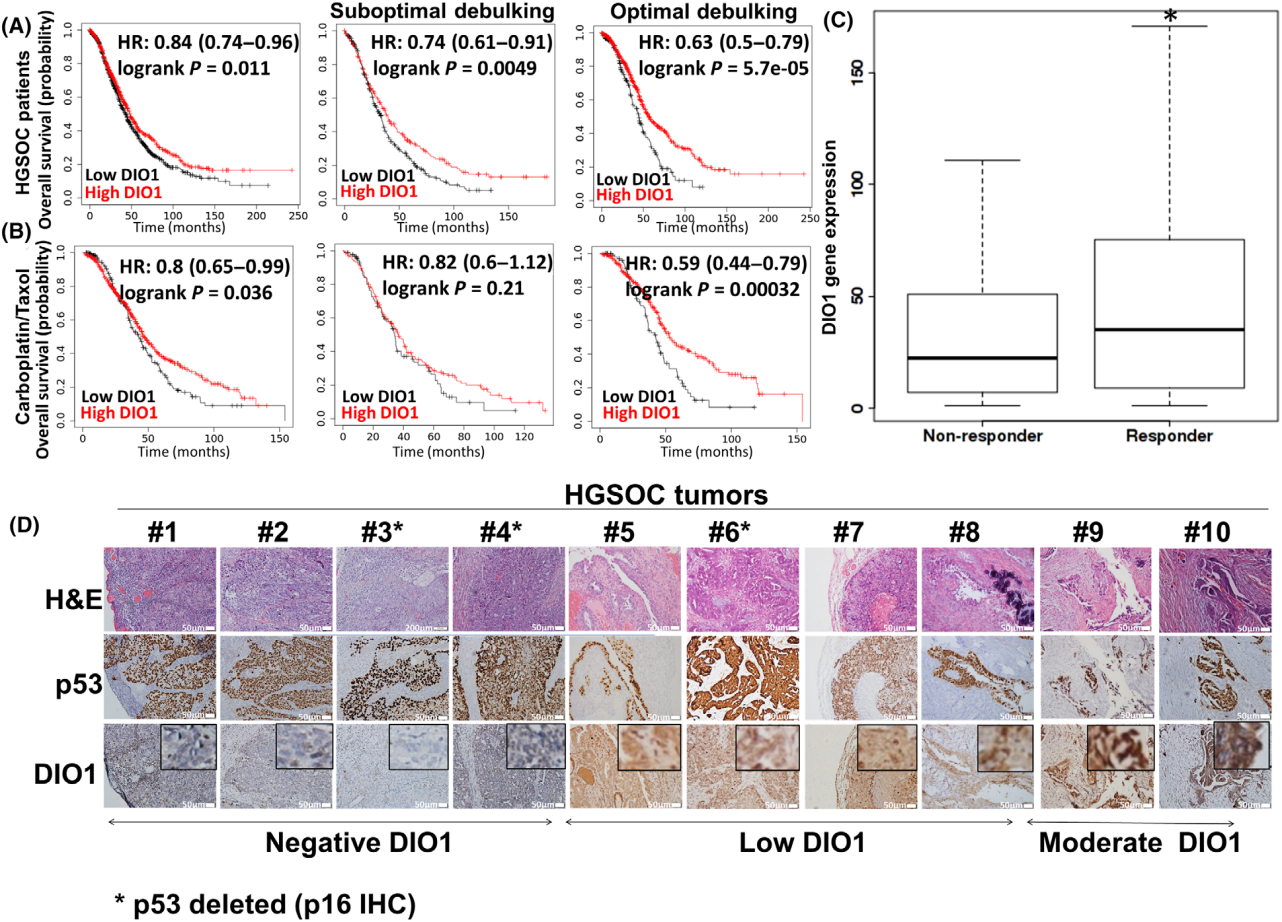
Selenoenzyme type I deiodinase (DIO1) correlates with overall survival in high‐grade serous ovarian cancer (HGSOC) patients. DIO1 expression (high vs. low) was analyzed in (A) HGSOC patients or (B) patients treated with carboplatin/taxol, who underwent optimal (no residual tumor) or suboptimal debulking surgery. Data were retrieved from The Cancer Genome Atlas (TCGA) using the Kaplan–Meier Plotter tool (http://kmplot.com). HR, hazard ratio. (C) Boxplots of DIO1 expression in responders vs. non‐responders, after therapy completion using the ROC Plotter (https://rocplot.com/custom‐data/index). *P value < 0.05 following Mann–Whitney test. (D) DIO1 immunohistochemistry (IHC) of formalin‐fixed paraffin‐embedded (FFPE) sections from human ovarian tumors (patients 1–10). Scale bar, 50 μm. Hematoxylin and eosin (H&E) and p53 staining were used to define tumor regions. p53 deleted cases (marked by an asterisk) underwent p16 staining. For each image, an enlarged inset of DIO1 expression is shown in the top right corner. Negative background stain was also confirmed using a matched isotype control antibody (Fig. S2B). 10× objective, Olympus microscopy (Olympus, Tokyo, Japan).

We explored the prognostic value of DIO1 using The Cancer Genome Atlas (TCGA) database and the Kaplan–Meier Plotter tool. Results (Fig. 2A) indicate that high DIO1 RNA expression correlates with better overall survival probability in HGSOC patients (hazard ratio, HR = 0.84, *P* = 0.011). This association was highly significant both in patients who underwent optimal (HR = 0.74, *P* = 0.0049) or suboptimal surgery (HR = 0.63, *P* = 5.7e‐05). Similarly, in HGSOC patients treated with the first‐line standard of care, carboplatin, and taxol (Fig. 2B), high DIO1 expression correlated with extended survival (HR = 0.8, *P* = 0.036) and more so following optimal debulking surgery (HR = 0.59, *P* = 0.00032). Analyses of DIO1 expression in serous subtype patients with optimal debulking surgery, after completing the therapy, using the ROCplot tool (Fig. 2C), further show that patients with complete pathological response (responders) had significantly higher DIO1 expression in comparison with patients with residual disease (non‐responders). These results suggest a benefit for HGSOC patients with high DIO1 expression and point toward a role for this enzyme as a potential tumor suppressor in this disease.

**Fig. 3:**
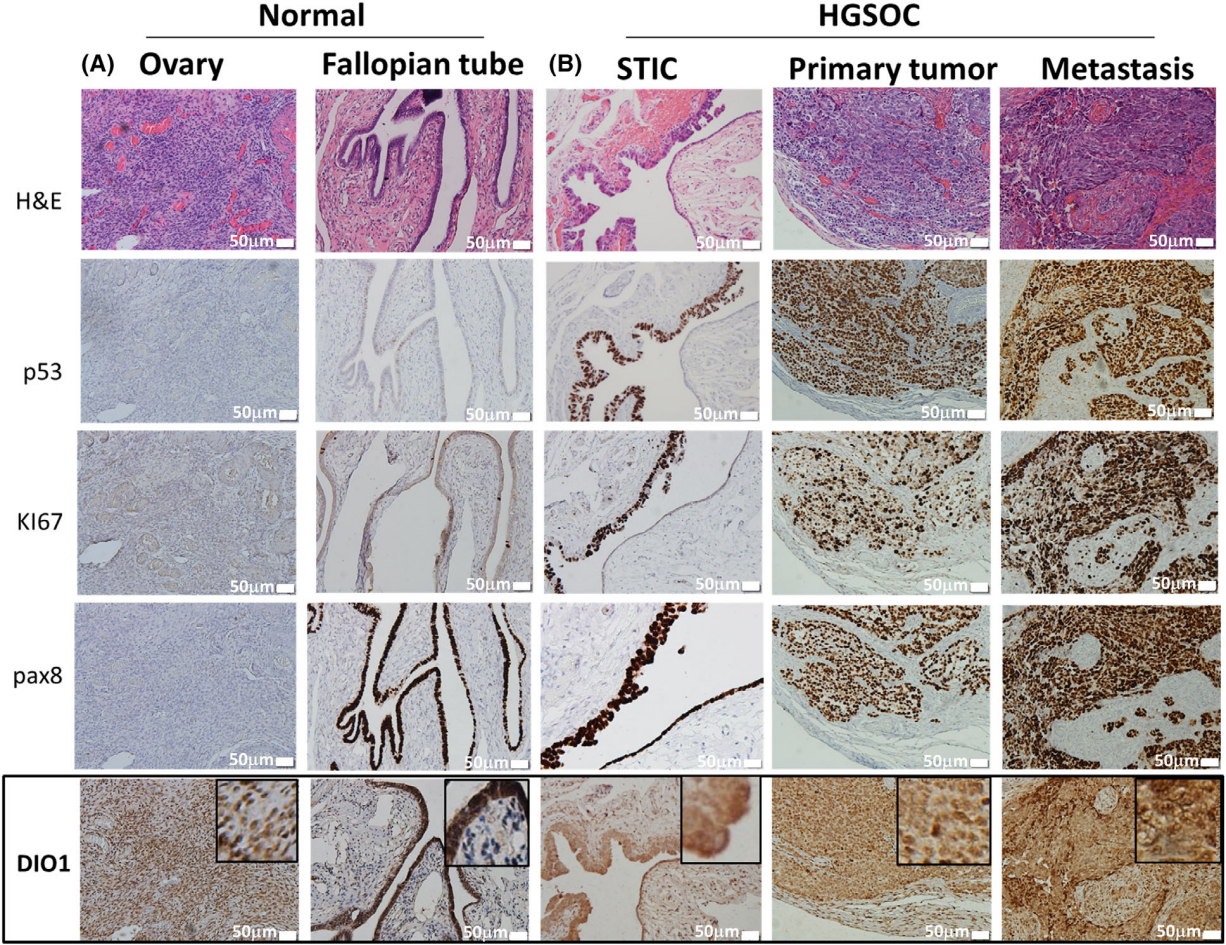
Selenoenzyme type I deiodinase (DIO1) expression in normal ovary, fallopian tubes, and throughout high‐grade serous ovarian cancer (HGSOC) evolution. Hematoxylin and eosin (H&E) and immunohistochemistry (IHC) analyses of p53, KI67, PAX8, and DIO1 in representative formalin‐fixed paraffin‐embedded (FFPE) tissues of (A) normal ovary and fallopian tubes and (B) serous tubal *in‐situ* carcinoma (STIC), primary and colon metastasis from a representative HGSOC patient (patient #7). Scale bar, 50 μm. 10× objective, Olympus microscopy (Olympus, Tokyo, Japan).

We next explored DIO1 protein expression in tissues from HGSOC human subjects. Age at diagnosis and patient's survival are presented in Table S2. Tumor sections from 10 patients underwent IHC analysis using DIO1 antibody. H&E and p53 or p16 staining were used to define tumor regions. Results indicate that DIO1 expression varied between patients, with four cases exhibiting negative staining, four low staining, and two presenting moderate expression in scattered tumor regions (Fig. 2D). Isotype control is presented in Fig. S2B. These results suggest heterogeneous DIO1 expression in HGSOC patients. We did not observe a correlation between DIO1 expression and patient's survival (*P* = 0.53), due to the small study size. Notably, the study cohort displayed homogenously high expression of DIO3, the T3‐catabolizing enzyme (Fig. S3). To study DIO1 expression pattern in normal tissues, ovary and fallopian tubes were collected from a total of eight non‐oncological patients (Fig. S4). Patients' age and diagnosis at tissue collection are presented in Table S2. All tissue slides were stained in parallel for H&E, p53, KI67, and pax8, a marker for secretory fallopian tube cells. Representative results (Fig. 3A) display background staining of DIO1 in normal ovaries, while in the serous of normal fallopian tubes, high DIO1 expression was evident. These results point toward a potential role of the DIO1 enzyme in the physiology of the fallopian tube. Lastly, we were interested to study DIO1 expression in the fallopian tube pre‐malignant regions (STIC), considered the disease site‐of‐origin. Representative STIC sections, following H&E, p53, KI67, and pax8 staining, show moderate DIO1 expression (Fig. 3B). This pattern remained the same in primary tumor, as well as metastatic site in the colon from the same patient. The limited expression of DIO1 throughout HGSOC evolution, from the early precursor FT lesion, to the primary and metastatic tumor sites, strongly implies that the enzyme downregulation may facilitate disease progression.

### 3.3 DIO1 expression contributes to ovarian cancer tumor suppression

**Fig. 4:**
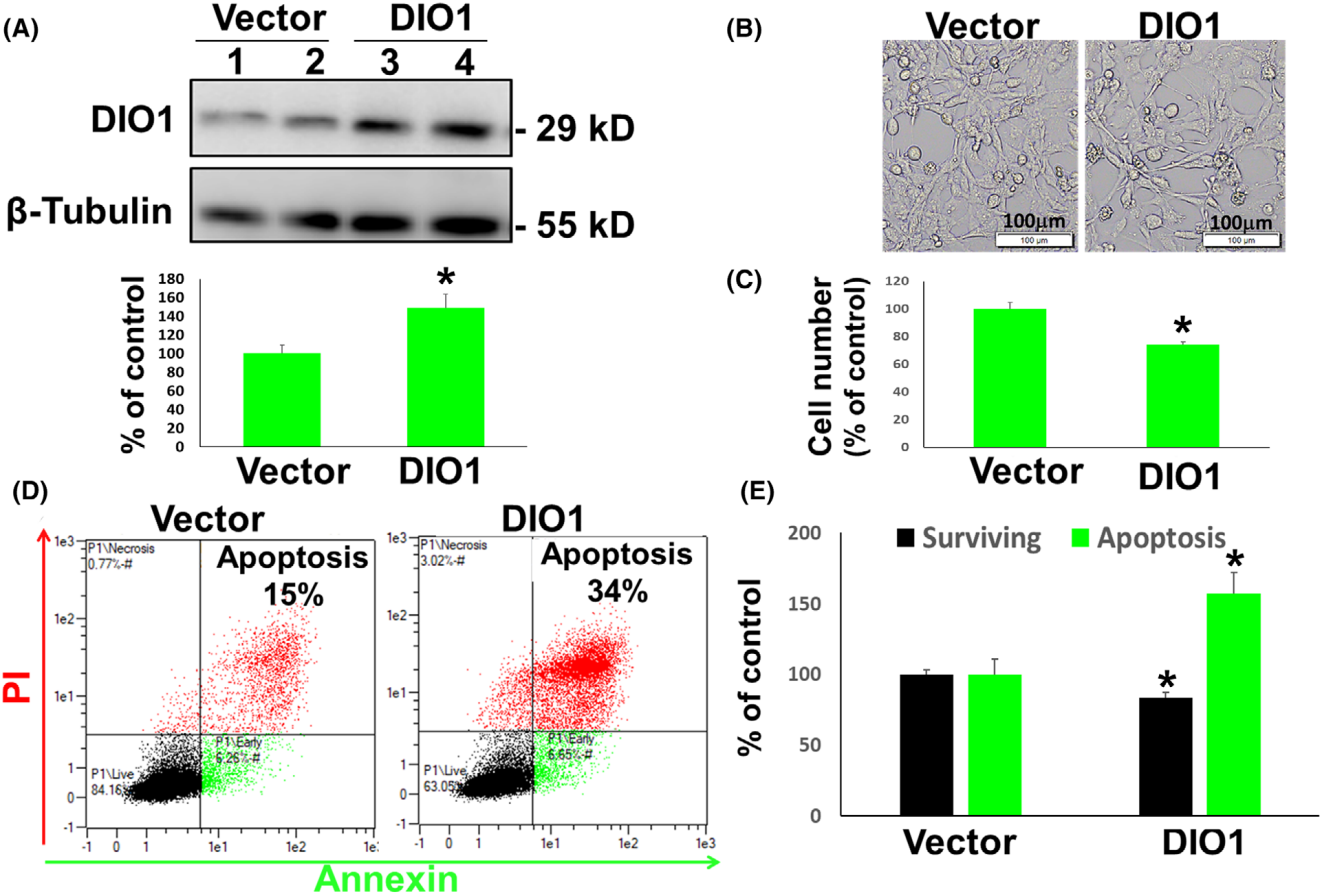
Selenoenzyme type I deiodinase (DIO1) overexpression inhibits high‐grade serous ovarian cancer (HGSOC) proliferation. ES‐2 cells transiently transfected with empty PCDNA3 or DIO1‐PCDNA3 expression vectors were analyzed after 3 days for (A) DIO1 protein levels in duplicates from control and DIO1 expression vectors are shown by Western blot (WB). β‐tubulin was used for protein loading. Average DIO1 protein expression ± SEM is shown in the lower panel. (B) Light microscopy (X10 images, scale bar, 100 μm). Olympus microscopy images, using Cell^A software imaging. (C) Absolute cell counts, FC. (D) Annexin‐PI histograms, FC. (E) Quantification of surviving cell population (Annexin−/PI−) and apoptotic cell population (Annexin+/PI+). Results (average ± SEM) were repeated twice, in triplicates. *P < 0.05 using Student's unpaired *t* test.

DIO1 diminished expression in high‐grade serous ovarian cancer, both *in vitro* and in human tissues, together with indications that high RNA expression positively correlates with patient's survival, led us to hypothesize that the enzyme may function as a tumor suppressor. To validate this assumption, we enhanced DIO1 protein levels using a DIO1 expression plasmid (DIO1‐PCDNA3). A selected HGSOC cell line (ES‐2) was transiently transfected with DIO1 expression vector or an empty vector and analyzed 3 days post‐transfection by several methods. First, induction of 40% in DIO1 protein levels was confirmed (Fig. 4A). This DIO1 overexpression resulted in lower cell density (Fig. 4B) and a 35% reduction in absolute cell counts (Fig. 4C), compared to control transfected cells. In parallel, DIO1 ectopic expression led to a significant apoptotic cell death, as shown by a representative flow cytometry Annexin‐PI histogram (Fig. 4D). Quantification of the cell populations shows an average reduction of 20% in cell survival and an induction of 65% in apoptotic cell death (Fig. 4E). These collective results established that DIO1 functions to suppress HGSOC cell growth.

**Fig. 5:**
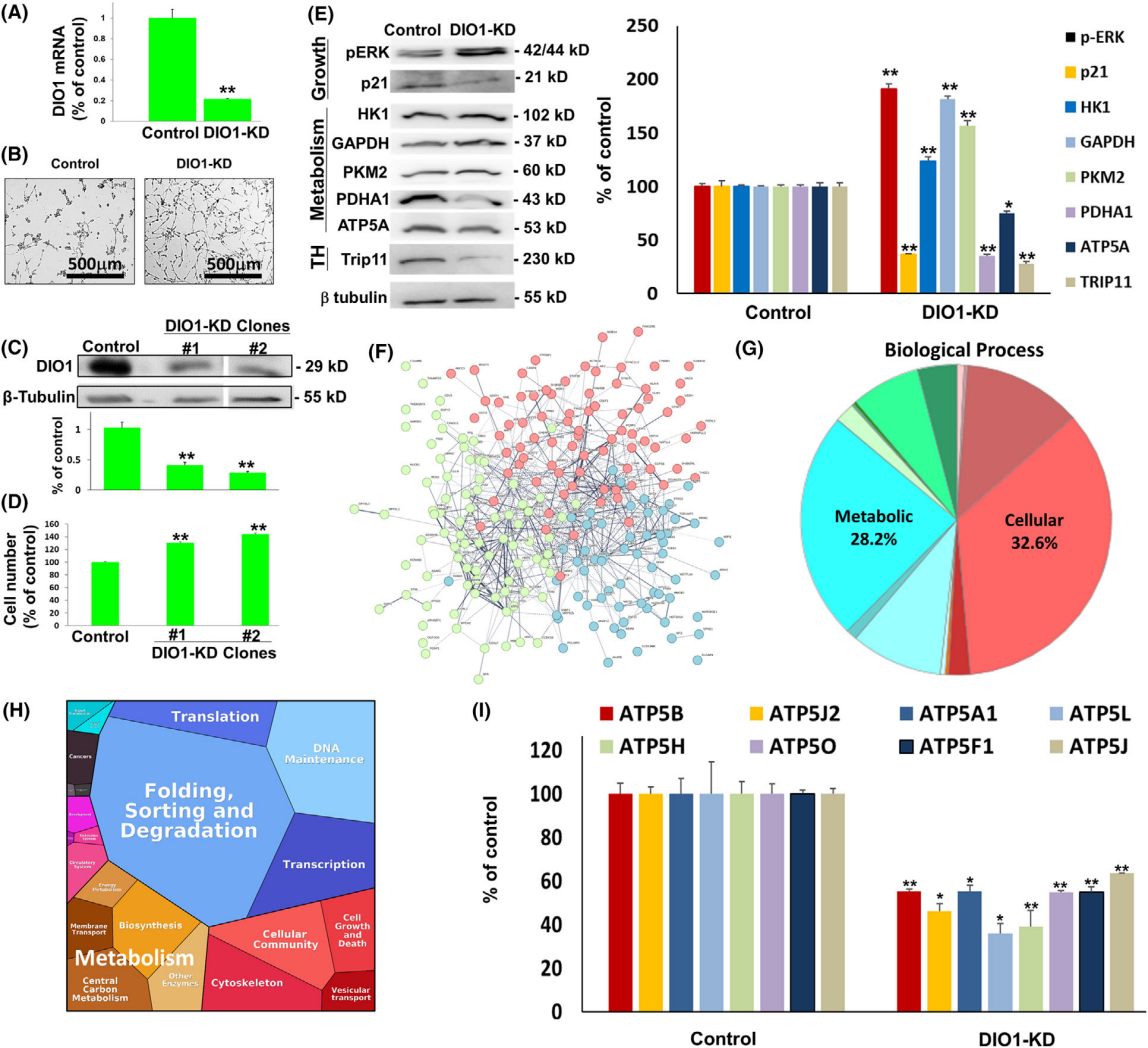
Selenoenzyme type I deiodinase (DIO1) silencing induces proliferation and alters protein expression in ovarian cancer. Analyses of DIO1 level in knockdown (KD) versus control ES‐2 cells for (A) mRNA transcription (RQ‐PCR). (B) Light microscopy of 1 × 10^5^ control and DIO1‐KD cells incubated in 24‐well plates for 96 h (×10 images, scale bar, 500 μm). Olympus microscopy images, using Cell^A software imaging. Isolated clones from DIO1‐KD versus control cells were analyzed for (C) DIO1 protein level by Western blot. (D) Cell number, by flow cytometry. (E) pERK, p21, HK1, GAPDH, PKM2, PDHA1, ATP5A, and Trip11 levels by Western blot. β‐tubulin was used for protein loading. (F) Proteomics analyses of differentially expressed proteins in DIO1‐KD high‐grade serous ovarian cancer (HGSOC). Interaction network, divided into three clusters, using string v. 10.5 (https://string‐db.org), with default settings (minimum required interaction score: medium confidence 0.4). (G) Pie chart using panther (http://pantherdb.org). Cellular and metabolic processes are shown. (H) Proteomaps using the bionic visualization tool (BionicVis). (I) Proteomics expression analyses for a collection of ATP5 synthase complex subunits in control and DIO1‐KD cells. Results (average ± SEM) are shown as % of control. Results were repeated twice in triplicates. *P < 0.05. **P < 0.005 by Student's unpaired *t* test.

Following the observation that overexpressing DIO1 leads to a reduction in HGSOC cell proliferation, we were interested to study whether silencing DIO1 would result in an opposite effect. We generated DIO1 knockdown ES‐2 cells (DIO1‐KD) using DIO1 shRNA plasmid. Scrambled shRNA served as a negative control. Using puromycin selection, we have generated stable DIO1‐KD ES‐2 cells with a 80% reduction (*P* < 0.005) in DIO1 mRNA levels (Fig. 5A). A significantly higher cell density was observed in the DIO1‐KD cells compared to cells transfected with the scrambled control (Fig. 5B). We isolated two DIO1‐KD clones in which a significant decrease in DIO1 protein levels was shown (Fig. 5C) and documented a comparable increase in cell number (Fig. 5D). The induced proliferation was accompanied by a significant increase in the mitogen‐related kinase, phosphorylated ERK (pERK), and a reduction in the cell cycle inhibitor p21 (Fig. 5E). In parallel, key glycolysis proteins, including hexokinase 1 (HK1), glyceraldehyde 3‐phosphate dehydrogenase (GAPDH), and pyruvate kinase 2 (PKM2), were induced. On the other hand, we observed a reduction in pyruvate dehydrogenase E1 subunit alpha 1 (PDHA1), the gate‐keeper enzyme which links between glycolysis and the citric acid cycle, as well as in ATP5A, which is part of the oxidative phosphorylation mitochondrial enzyme complex (ATP5) that provides cellular energy through the synthesis of ATP. These metabolic changes are linked with fueling cancer cell proliferation via aerobic glycolysis (Warburg effect) [22]. In parallel, indirect evidence that DIO1 silencing hindered endogenous T3 levels was provided by the downregulation in TRIP11, a T3‐regulated protein which depends on this hormone for its interaction with the thyroid hormone nuclear receptor.

To better comprehend the global effects of DIO1 expression and the cell phenotype in ovarian cancer following DIO1 silencing, we performed proteomic analysis. For that, proteins were extracted from control and DIO1‐KD ES‐2 cells and analyzed in duplicates by reversed‐phase capillary chromatography coupled online to tandem mass spectrometry (uLC‐MS/MS). From the full list of differentially expressed proteins in the DIO1‐KD cells, we set a threshold of 1.25‐fold change from control transfected cells. This resulted in a total of 101 upregulated proteins and 123 downregulated proteins (Table 1). Interaction network, generated using the string tool (Fig. 5F), indicated an enrichment *P*‐value < 1.0e‐16, suggesting that the proteins are biologically connected, as a group. Functional analysis using panther revealed that reduction of DIO1 expression affected proteins involved in central biological processes (Fig. 5G). The main affected pathways were linked with cellular process (32.6%), as well as metabolic process (28.2%). In accordance with the panther results, the Bionic visualization (BionicVis) tool (Fig. 5H) confirmed alteration in various biological processes, including metabolism. Within this specific biological function, a cluster of eight proteins of the mitochondrial ATP5 complex, including ATP5A which was confirmed by Western blot (Fig. 5E), were significantly reduced by 36–61% following DIO1 silencing (Fig. 5I). This, together with a 31% reduction in another mitochondrial ATP synthase protein, USMG5, as well as a complete eradication of cytochrome c oxidase assembly factor 6 (COA6), which encodes an assembly factor for mitochondrial complex IV, suggests a direct role for DIO1 in mitochondrial energy production. We further observed under DIO1 depletion upregulation in an array of proteins involved mainly in cancer cell proliferation. These include two A‐kinase anchoring protein family members (AKAP8 and AKAP12), DNA primase polypeptide 1 (PRIM1), and two Cullin 4 proteins (CUL4A and CUL4B), which were upregulated following DIO1‐KD. Another mechanistic rationale for the induction in cell proliferation upon DIO1 silencing is the 27% increase in another selenoprotein family member, thioredoxin reductase 1 (TrxR1, encoded in human by the TXNRD1 gene). This protein is a key enzyme for protection against oxidative stress and was also reported to promote cancer progression in various tumor types, including HGSOC [23]. These global changes in protein expressions upon DIO1 silencing provide mechanistic explanation for the proliferative phenotype in ovarian cancer cells. For a selected set of proteins, including CUL4 and TrxR1, the proteomics results were validated by Western blots (Fig. S5).

**Table 1:** Altered proteins in DIO1‐KD ovarian cancer.

Downregulated proteins			Upregulated proteins		
Name	% of ctrl	*P* value	Name	% of ctrl	*P* value
COA6	−100	0.000031	AKAP8	Unique	0.000042
STX6	−100	0.000158	PRIM1	Unique	0.000102
FDFT1	−100	0.000184	MRPS25	Unique	0.000330
BCKDHB	−100	0.000360	ZNF22	Unique	0.000593
SUZ12	−100	0.000660	CUL4A	Unique	0.000650
OGFOD3	−100	0.000769	HMGN3	950	0.000693
STAT5B	−100	0.000885	DAB2	126	0.000944
METTL14	−100	0.000964	ACOX1	106	0.000995
ASAH1	−100	0.001083	AKAP12	98	0.001009
HINT3	−100	0.001181	UACA	93	0.001039
GSTZ1	−100	0.001208	GOSR2	89	0.001257
DLGAP4	−100	0.001261	UBE2A	82	0.001343
RRAGC	−100	0.001283	ADD1	76	0.002215
PIH1D1	−100	0.001298	CISD1	74	0.002891
MTIF2	−100	0.001324	SERPINB2	72	0.002920
RIPK1	−100	0.001525	GOPC	66	0.003091
RBBP9	−100	0.001666	PRIM2	66	0.042453
EDC3	−100	0.001981	ANO10	65	0.003442
NTPCR	−100	0.002060	ARHGAP29	63	0.003640
LDLR	−100	0.002133	DYNLT1	61	0.003703
GNE	−100	0.002177	LMNA	61	0.004057
CCDC88B	−100	0.002201	CBX5	60	0.004707
DNM1	−94	0.002215	ECI2	59	0.004853
MOCOS	−86	0.002455	ACO1	58	0.005437
UBXN7	−84	0.002720	MTMR2	57	0.005442
SCAMP1	−76	0.003167	AASS	57	0.005709
LAMC1	−68	0.003196	DPYSL3	57	0.005722
TMEM167A	−64	0.003271	ELOVL5	56	0.005865
ATP5H	−61	0.003347	PGM3	56	0.006833
FABP5	−60	0.003497	PIGS	53	0.006955
FKBP1A	−59	0.004066	EIF1AX	52	0.007210
LOX	−58	0.004087	MSH6	51	0.007260
CHID1	−58	0.004218	NUCB1	51	0.007364
CTSZ	−56	0.004297	AGPS	51	0.007609
ITPA	−56	0.004517	POLDIP3	49	0.007722
LRRC20	−55	0.005037	PFDN1	48	0.008037
ATP5J2	−54	0.005082	SLBP	47	0.008877
TGM2	−54	0.005382	CLASP1	47	0.009058
RCN1	−54	0.006116	TOP2B	46	0.009387
ADNP	−52	0.006228	PQBP1	46	0.010255
CNDP2	−51	0.006574	UBA1	45	0.010351
FAM162A	−51	0.006691	FHOD1	44	0.010584
AIP	−51	0.006781	PSMD10	44	0.010593
LMAN1	−51	0.006873	PDCD6IP	43	0.010665
THOC1	−50	0.006915	ETHE1	43	0.010929
C11orf68	−49	0.007027	LOXL2	43	0.011197
POFUT1	−48	0.007239	AARSD1	43	0.011728
RIOX2	−48	0.007354	BZW1	43	0.011833
AMOTL2	−48	0.007402	STAG2	43	0.011852
CSRP1	−48	0.007955	HSPA1B	42	0.011914
WDR6	−47	0.008191	AGFG1	42	0.012025
APOOL	−47	0.008298	HSPA13	42	0.012112
NIT2	−47	0.008337	CD99	41	0.012835
NARS	−47	0.008576	PPFIBP1	40	0.012847
ARFIP2	−46	0.008808	DYNC1LI1	40	0.013569
ATP5O	−45	0.009201	HLA‐A	40	0.013595
ATP5F1	−45	0.009316	HLA‐A	39	0.013606
ATP5B	−45	0.009324	TUBB	38	0.014768
ATP5A1	−45	0.009649	MPDU1	38	0.014788
PIGT	−43	0.010474	CLIP1	38	0.014789
ATOX1;ATO	−43	0.010475	TXN	38	0.015024
GALE	−42	0.010545	PSMB6	37	0.015139
PAPSS1	−40	0.010929	LSM8	36	0.015280
GOLIM4	−40	0.011239	CTSC	36	0.015357
HYOU1	−40	0.011286	CUL4B	35	0.015417
DTD1	−39	0.011323	MARCKSL1	35	0.016278
ARIH2	−39	0.011366	PPP2R5D	34	0.016300
MGST3	−39	0.011594	PURA	34	0.016447
COX17	−38	0.011651	PPAN	34	0.016551
PKM	−38	0.012138	TRIM25	34	0.016683
RHOG	−38	0.012264	HIST1H1A	33	0.016791
RNH1	−37	0.012474	LPCAT1	33	0.016827
GALNT2	−37	0.012780	RBM42	33	0.016865
ATP5J	−36	0.012797	COPS7A	32	0.017127
P4HA1	−36	0.012917	THUMPD3	32	0.017623
MANF	−36	0.013198	PHAX	31	0.017971
CDV3	−35	0.013344	RAB8A	31	0.018013
CTSD	−35	0.013619	SH3KBP1	31	0.018493
SCD	−35	0.013735	SLC25A6	31	0.018630
USP14	−34	0.013782	S100A16	31	0.018693
NAT10	−34	0.014353	VCL	31	0.019432
SND1	−33	0.016384	NT5DC1	30	0.019575
HCLS1	−33	0.017317	CYCS	29	0.019621
FLNC	−33	0.017450	STAM2	29	0.019901
EIF2A	−33	0.017467	GGCX	29	0.019941
APMAP	−32	0.017614	ASMTL	29	0.020037
NUDCD1	−32	0.017701	MTHFD1L	29	0.020651
CALR	−32	0.018336	MTCH1	29	0.021190
TPD52L2	−32	0.018509	ARHGEF1	28	0.021572
GBE1	−31	0.018861	MOB1A	28	0.021700
CCDC58	−31	0.018963	FLNA	28	0.021894
POLR2H	−31	0.019988	CAV1	27	0.021992
USMG5	−31	0.020401	TRIR	27	0.022062
GMPS	−31	0.021020	TXNRD1	27	0.022239
SEC61B	−31	0.021450	COPS6	27	0.022396
TOR1AIP2	−31	0.021526	UGDH	26	0.022490
SH3BGRL	−30	0.021768	NF2	25	0.022618
CPNE1	−30	0.022055	DST	25	0.022622
ACTL6A	−29	0.022140	ACTR3	25	0.022761
PLOD2	−29	0.022349	SEPT9	25	0.022860
VPS4B	−29	0.022534	ILKAP	25	0.024563
PAK2	−29	0.022698			
PSMD2	−29	0.022717			
FAM49B	−28	0.022824			
DCUN1D1	−28	0.023256			
PACSIN3	−28	0.023318			
CORO1B	−28	0.024100			
PODXL	−28	0.024443			
PTPN23	−27	0.024503			
HSP90B1	−27	0.024606			
KTI12	−27	0.024705			
LMAN2	−27	0.024823			
GSTP1	−27	0.025042			
MAGED2	−26	0.025469			
IARS2	−26	0.025625			
RRAS2	−25	0.026020			
TOMM70	−25	0.026092			
CSTF1	−25	0.026103			
RAB7A	−25	0.026460			
ARL6IP5	−25	0.026560			
PPP1R14B	−25	0.027171			
ACBD3	−25	0.027590			
FKBP2	−25	0.027901			

Unique/protein identified exclusively in the DIO1‐KD cells.

**Fig. 6:**
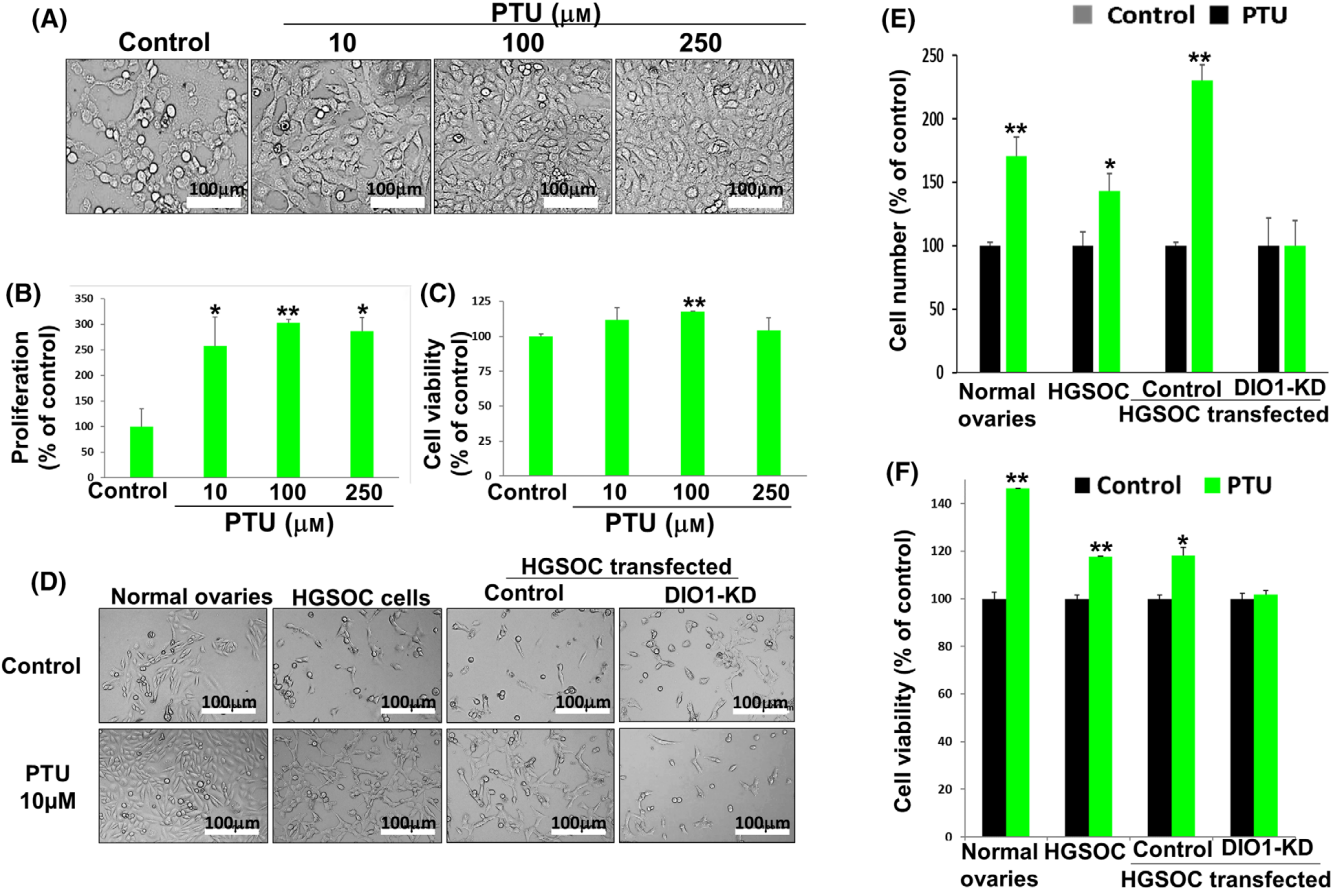
Selenoenzyme type I deiodinase (DIO1) inhibition promotes ovarian cancer cell growth. ES‐2 cells were seeded (1000 cells/96 wells), treated with PTU at increasing concentrations, and analyzed after 96 h for (A) cell density by light microscopy (×10 images, scale bar, 100 μm). Olympus microscopy images, using Cell^A software imaging. (B) Cell proliferation (CyQUANT, ELISA). (C) Cell viability (PrestoBlue, ELISA). Next, normal ovary cells (CHO‐K1), HGSOC cells (ES‐2), and scrambled and DIO1‐KD ES‐2 cells were treated with 10 μm propylthiouracil (PTU) and analyzed by (D) Light microscopy (×10 images, scale bar, 100 μm). Olympus microscopy images, using Cell^A software imaging. (E) Absolute cell counts (flow cytometry). (F) Cell viability (PrestoBlue, ELISA). Results (average ± SEM) were repeated twice in triplicates. *P < 0.05. **P < 0.005 using ANOVA.

Lastly, we wanted to establish, by an additional approach, that DIO1 inhibition contributes to tumor progression. To that end, we used propylthiouracil (PTU), a well‐established non‐competitive selective DIO1 inhibitor [24]. The HGSOC cells, ES‐2, were seeded (1000 cells/96 wells), treated with increasing PTU concentrations (10–250 μm), and analyzed after 4 days by an array of complementary methods. Microscopy results showed an increase in cell density following PTU treatments (Fig. 6A). These results were confirmed by a significant increase of 2.5‐ to 3‐fold in cell proliferation (Fig. 6B), accompanied by a minor increase in cell viability, which reached statistical significance only at 100 μm PTU (Fig. 6C). In order to establish that the effect of PTU on cell proliferation was DIO1‐mediated, we combined the two approaches of DIO1 inhibition, the use of the selective inhibitor (PTU) with DIO1‐silencing (DIO1‐KD). We treated control and ES‐2 DIO1‐KD cells with PTU (10 μm) for 96 h. Normal ovarian cells (CHO‐K1) and the parental ES‐2 cells, both expressing endogenous DIO1 protein, were used for comparison. Figure 6D depicts that in ES‐2 cells transfected with control shRNA, addition of PTU induced cell density, comparable to the results obtained in the parental ES‐2 cells and normal ovarian cells. In the DIO1‐KD cells, however, the effect of PTU was prevented. Under the same experimental settings, comparable results were shown for cell proliferation (Fig. 6E) and cell viability (Fig. 6F). Collectively, our data established that inhibition of DIO1 led to proliferation induction in HGSOC cells, suggesting that this enzyme functions as an anti‐proliferative protein in these cells. This effect is directly mediated via DIO1, as no effect was shown by PTU in the absence of this enzyme.

## 4 Discussion

In this work, we have established that the expression of DIO1 is attenuated in HGSOC cells compared to normal epithelial cells. DIO1 protein expression in human HGSOC tumors ranged from negative to moderate, suggesting heterogeneity between patients. We have further demonstrated that the expression of DIO1 is maintained at a relatively low level, not only in the primary and metastatic tumor sites but also in the pre‐malignant fallopian tube regions. These results strongly suggest that restricted DIO1 expression may be an early event in the evolution of HGSOC. Our observation that DIO1 expression is disturbed is supported by previous reports in different types of cancer, with a similar reduced expression in papillary thyroid carcinoma [25, 26, 27, 28, 29], thyroid adenoma [30], lung cancer [31], hepatic cancer [32, 33], and clear cell renal cell carcinoma [34, 35, 36, 37, 38]. This collective evidence proposes that DIO1 reduction in cancer may be a common phenotype, implying a mechanistic role.

In order to better understand the possible function of DIO1 in HGSOC, we designed experiments in which DIO1 was overexpressed. Our studies indicated that DIO1 induction in ovarian cancer cells attenuates proliferation and tumor growth. These results are comparable with studies performed in renal cell carcinoma [37, 38] in which, similar to our experiments, downregulation of proliferation following ectopic DIO1 expression was documented. These tumor‐inhibition outcomes in renal cancer models were suggested to be mediated via deiodination of T4 and production of T3, a powerful regulator of cellular differentiation.

After observing that DIO1 acts to attenuate ovarian cancer cell proliferation, together with its decreased expression in HGSOC compared to normal cells, we examined the outcome of lowering this protein by direct catalytic inhibition or silencing the DIO1 enzyme. Our results indicate that both approaches led to cell proliferation. These results are further supported by TCGA data analysis, which indicated that HGSOC patients with a low expression of DIO1 mRNA exhibit shorter survival compared to patients with high DIO1 levels. It should be noted that, due to the small study cohort, we were unable to present a similar correlation at the protein level.

The molecular mechanisms responsible for the tumor promoting phenotype following DIO1 silencing were elucidated by changes in signaling pathways regulating cell proliferation, including a significant elevation in pERK and a reduction in the cell cycle inhibitor p21. In accordance with the induced proliferation, increase in two AKAP family members [39, 40], and two CUL4 proteins [41, 42], reported to promote the progression of various tumors, including that of the ovary. Moreover, PRIM1 was completely absent in our control transfected cells and was upregulated following DIO1‐KD. This protein is overexpressed in highly aggressive ovarian cancer cell lines [43] and is directly involved with cell proliferation in breast and colon cancers [44, 45]. Interestingly, PRIM1 was shown, in a brain‐development model, to be a repressed thyroid‐hormone responsive gene [46], providing an indirect indication for depleted T3 levels following DIO1 silencing.

DIO1 reduction led to an increase in HK1, GAPDH, and PKM2, indicating a metabolic shift toward glycolysis, also known as the Warburg effect [22]. Similarly, TrxR1, an enzyme which is essential for redox homeostasis and is a major regulator of glycolysis [47], was induced upon DIO1 silencing. TrxR1 is overexpressed in an array of cancer types and is considered a therapeutic target in ovarian cancer [23]. Notably, similar to DIO1, TrxR1 is also a member of the selenoprotein family. In parallel, a cluster of eight ATP5 subunits were attenuated. Such downregulation indicates attenuation in mitochondrial electron chain activity, resulting in a reduced oxidative phosphorylation [48] and was linked with tumor progression in several tumors [49, 50, 51, 52, 53]. A similar reduction was documented in PDHA1, a critical component of the pyruvate dehydrogenase complex and a rate‐limiting step in the transformation of pyruvate into acetyl‐CoA and entry into the TCA cycle for producing ATP via oxidative phosphorylation. In cancer, PDHA1 is considered a central factor regulating the metabolic switch from oxidative phosphorylation to aerobic glycolysis. Inhibition of PDHA1 has been shown to decrease mitochondrial OXPHOS and promote tumor aerobic glycolysis in tumor cells, while an opposite effect was reported for PDHA1 overexpression [54, 55]. These collective metabolic changes observed in our DIO1‐KD cells correlate with enhanced proliferation and highlight that the absence of DIO1 enzyme produces an environment which attenuates oxidative phosphorylation and intensifies the Warburg effect.

The biologically active thyroid hormone T3 is transcription factor and a major endocrine regulator of metabolic rate, with profound impact on mitochondrial ATP production [56]. In fact, several of the ATP5 subunits which were reduced in our study were reported to be directly upregulated by T3 in the oncological and non‐oncological context [57, 58, 59]. Another T3‐target gene that belongs to the ATP5 family member and was reduced in our current study is USMG5, a mitochondrial ATP synthase [60]. Lastly, both TRIP11 [61] and PKM2 [62] are also known to be T3‐regulated. These results suggest that the changes observed in DIO1‐depleted cells may be due to reduced intracellular T3. Support for this notion comes from work by our group on another deiodinase family member, DIO3, which catabolizes T3. Reduction in DIO3 resulted in upregulation of the same set of ATP5 proteins [14], parallel to inhibition of proliferation [14, 63]. Moreover, DIO3‐KD led to a reduction in pERK and PKM2 and an elevation in TRIP11. The fact that DIO1 and DIO3 silencing led to opposing outcomes on both cell proliferation and the same set of proteins, some of which direct T3‐target genes, suggests that these effects may be mediated via differential regulation of intracellular thyroid hormone levels. This assumption, however, should be corroborated by additional experiments.

## 5 Conclusions

In summary, we demonstrate the first indication in ovarian cancer that DIO1 is an anti‐tumor factor in the carcinogenic process, suggesting that reduction in its expression, consistently observed in several HGSOC cell lines and human tissues, may promote tumorigenesis in this aggressive disease.

## Conflict of interest

The authors declare no conflict of interest.

## Author contributions

AA and DM preformed the cell‐based experiments. AW, AK, and DK assisted in the IHC assays and analyses. MB, DJ, AUA, YH, YY, and OW collected the patients' tissues. ME and GI reviewed and analyzed the manuscript. OA‐F designed, analyzed, and interpreted the experimental data and wrote the manuscript. All authors read and approved the manuscript.

## Supporting information


**Fig. S1.** Specificity of the DIO1 antibody.
**Fig. S2.** Representative isotype control IHC staining.
**Fig. S3.** DIO3 expression in tumors from the study cohort.
**Fig. S4.** DIO1 expression in normal ovary and fallopian tubes.
**Fig. S5.** Validation of selected proteomics results by Western blot.

**Table S1.** Antibody list.
**Table S2.** Study cohort data.

## Data Availability

Full proteomics datasets will be available from the corresponding author upon a reasonable request.
