# Supplementary material for: The selenoenzyme type I iodothyronine deiodinase: a new tumor suppressor in ovarian cancer
Source: Mol Oncol. 2024 Mar 1;18(9):2298–313. doi: 10.1002/1878-0261.13612 (PMC11467794; doi:10.1002/1878-0261.13612)

**Supplemental data**

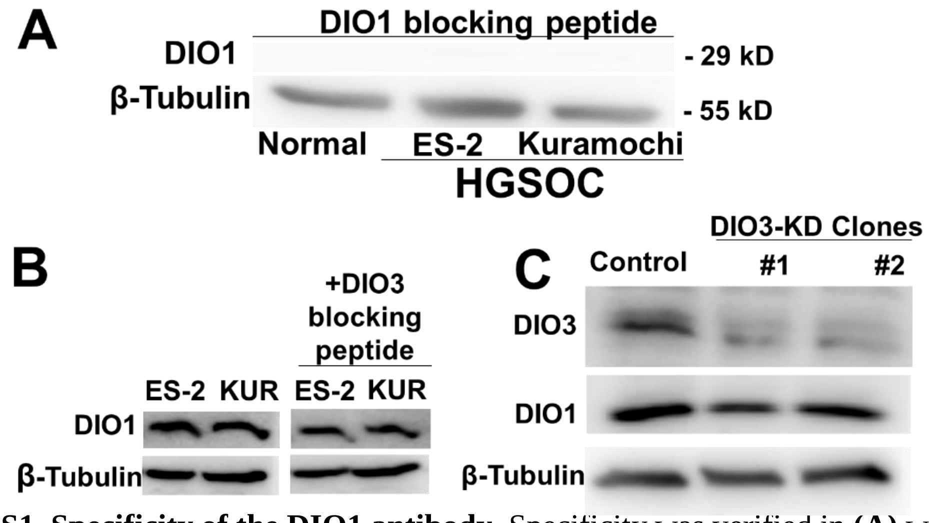


**Figure S1. Specificity of the DIO1 antibody.** Specificity was verified in **(A)** whole-cell lysates of normal ovaries (CHO-K1) and HGSOC cells (ES-2 and KURAMOCHI) co-incubated with DIO1 blocking peptide and examined for DIO1 levels **(B)** Whole-cell lysates of ES-2 and KURAMOCHI (KUR) co-incubated with DIO3 blocking peptide and examined for DIO1 levels by Western blots **(C)** Total lysates of control and DIO3-KD cells were examined for DIO1 and DIO3 levels by Western Blots**.** Tubulin was used as loading control.

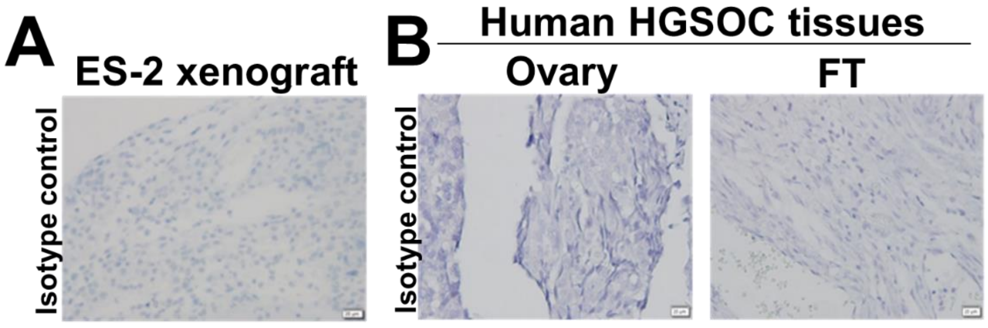


**Figure S2. Representative isotype control IHC staining** in **(A)** ES-2 cells and **(B)** ovary and FT human tissues (20X magnification, scale bars: 20 µm)

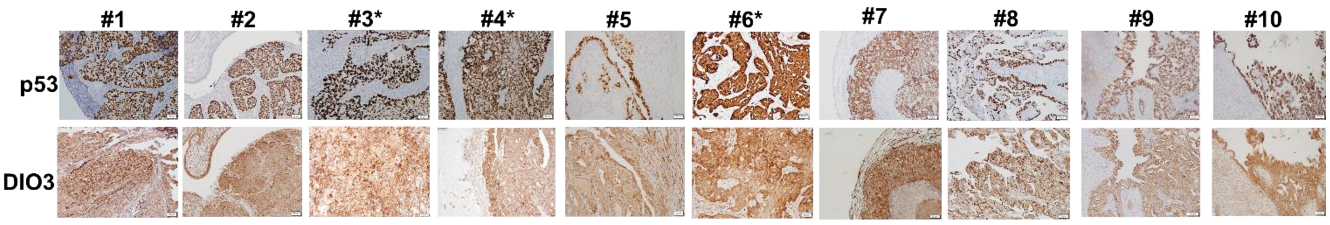


**Figure S3: DIO3 expression in tumors from the study cohort.** DIO3 IHC of FFPE sections in tumor sections from ten HGSOC patients. In all tissues p53 staining, or p16 (in p53-deleted cases, marked by *), were done to identify tumor regions. 10X objective, Olympus microscopy (Olympus, Tokyo, Japan).

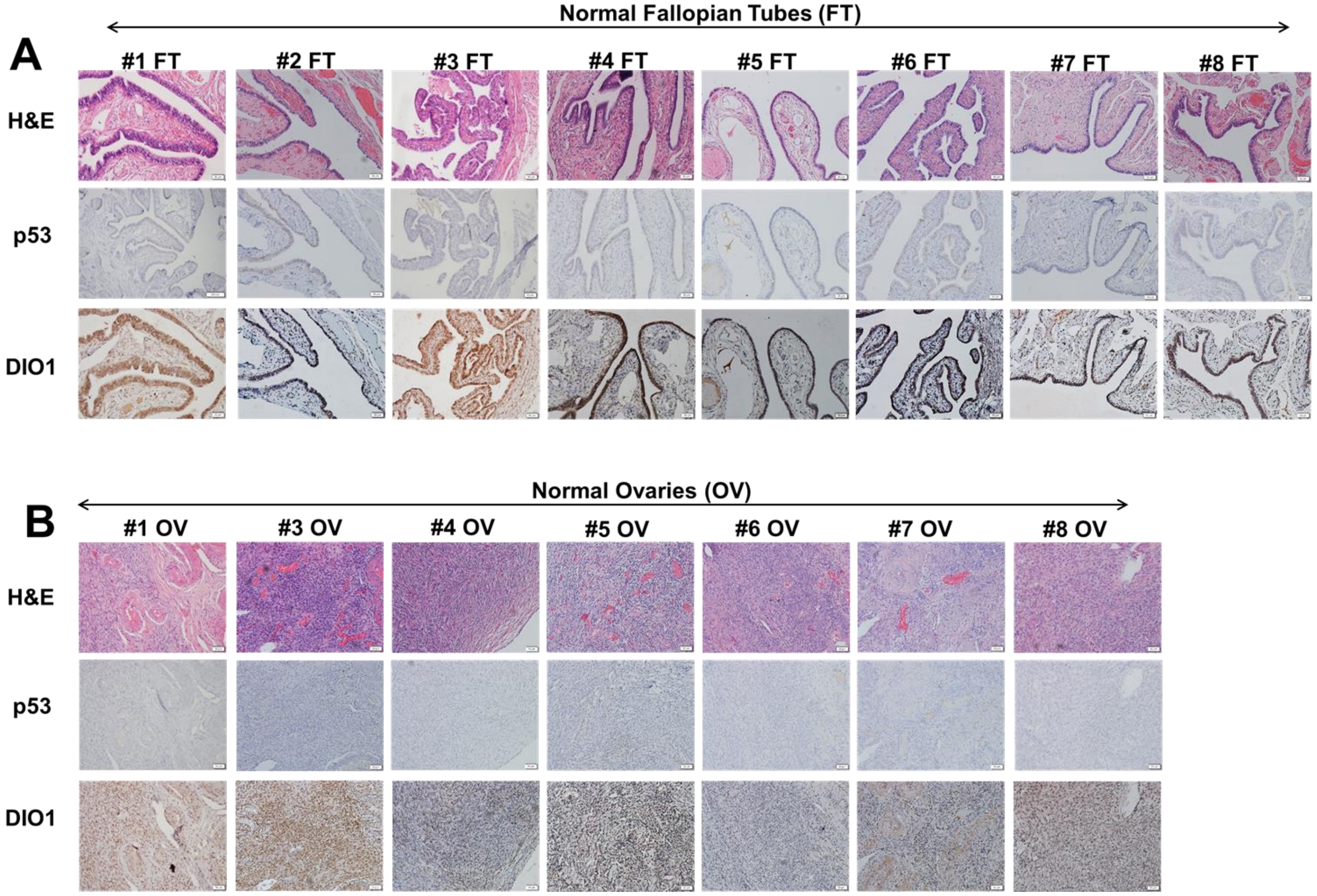


**Figure S4. DIO1 expression in normal ovary and fallopian tubes.** IHC of FFPE sections in **(A)** normal ovary and **(B)** fallopian tube from eight non-oncological patients. In all tissues H&E and negative p53 staining were used to exclude tumor regions. 10X objective, Olympus microscopy (Olympus, Tokyo, Japan).

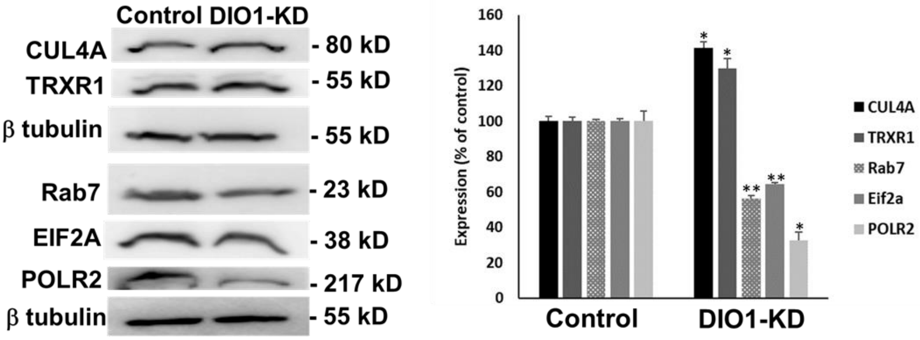


**Figure S5. Validation of proteomics results.** A selected set of altered proteins were examined using Western blots on whole-cell lysates of control and DIO1-KD ES-2 cells**.** Tubulin was used as loading control.

**Uncropped blot for Figure 4A:**

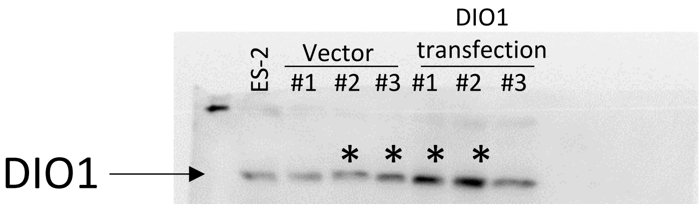


**Uncropped blot for Figure 5C:**

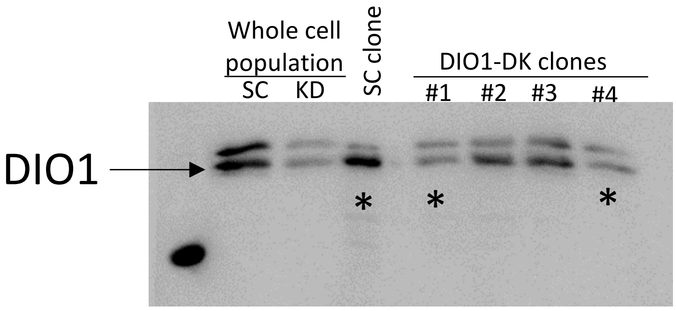

Supplement: Supplementary file 1 — Fig. S1. Specificity of the DIO1 antibody. Fig. S2. Representative isotype control IHC staining. Fig. S3. DIO3 expression in tumors from the study cohort. Fig. S4. DIO1 expression in normal ovary and fallopian tubes. Fig. S5. Validation of selected proteomics results by Western blot. [file MOL2-18-2298-s001.pdf]
